# Intraoperative Intermittent Pneumatic Compression Reduces Incidence of Venous Thromboembolism in Patients Undergoing Craniotomy: Study Protocol of a Randomized Multicenter, Single-Blind Trial

**DOI:** 10.1227/neuprac.0000000000000109

**Published:** 2024-08-28

**Authors:** Maximilian Scheer, Grit Schenk, Bettina Taute, Michael Richter, Michael Hlavac, Jens Gempt, Matthias Krammer, Ehab Shiban, Michael Sabel, Marco Stein, Andreas Wienke, Anke Höllig, Christian Strauss, Stefan Rampp, Julian Prell

**Affiliations:** * Department of Neurosurgery, University Hospital Halle, Halle, Germany;; ‡ Department of Internal Medicine, Angiology Division, University Hospital Halle, Halle, Germany;; § Coordination Center for Clinical Trials, University of Halle, Halle, Germany;; ‖ Department of Neurosurgery, Bezirksklinikum Günzburg, University of Ulm, Ulm, Germany;; ¶ Department of Neurosurgery, University Hopsital Hamburg-Eppendorf, Hamburg, Germany;; # Department of Neurosurgery, Klinikum Bogenhausen, München, Germany;; ** Department of Neurosurgery, Carl-Thiem-Klinikum Cottbus, Cottbus, Germany;; ‡‡ Department of Neurosurgery, University Hospital Düsseldorf, Düsseldorf, Germany;; §§ Department of Neurosurgery, University Hospital Gießen, Giessen, Germany;; ‖‖ Institute of Medical Epidemiology, Biostatistics, and Informatics, University of Halle-Wittenberg, Halle, Germany;; ¶¶ Department of Neurosurgery, Department of Neuroradiology, University Hospital Erlangen, Erlangen, Germany;; ## Department of Neurosurgery, University Hospital Aachen, Aachen, Germany

**Keywords:** Deep vein thrombosis, Pulmonary embolism, Neuro-oncology

## Abstract

**BACKGROUND AND OBJECTIVE:**

Venous thromboembolism (VTE), which includes deep vein thrombosis (DVT) and pulmonary embolism (PE), is a common complication in craniotomy patients. The duration of surgery has been identified as a risk factor for the development of VTE. In a pilot study, the use of intermittent pneumatic venous compression (IPC) dramatically reduced the incidence of VTE. Despite randomization, a significant difference in the duration of surgery between the groups limited the validity of this result. The study was underpowered to compensate for this problem. We now present the protocol of a multicenter trial.

**METHODS:**

All patients receive medical compression stockings and low-molecular-weight heparin from the first postoperative day. The therapy group receives IPC stockings intraoperatively. Postoperatively, all patients receive lower-extremity duplex sonography to detect/exclude DVT within the first 7 postoperative days. Contrast-enhanced chest CT is the gold standard for the detection of PE and is performed in cases of clinical suspicion of PE.

**EXPECTED OUTCOMES:**

The incidence of VTE is the primary end point. The distinction between symptomatic and asymptomatic, etiologies, influence of lesion type, duration of surgery, and mortality will be evaluated as secondary end points. The pilot study showed a VTE incidence of 26% in the control group vs 7% in the treatment group. To avoid overly optimistic treatment effect assumptions, we assume VTE rates of 9% and 24% in the treatment and control groups, respectively, and thus calculated a number of 127 patients per treatment group.

**DISCUSSION:**

If this trial shows that intraoperative IPC reduces the risk of VTE to the extent observed in our pilot study (number needed to treat: 5.24), the potential benefit to neurosurgical patients would be significant. The results would potentially influence treatment guidelines by providing the high-quality evidence needed to make robust recommendations.

**ABBREVIATIONS:**
DRKS: Deutsches Register Klinischer Studien
DVT: deep vein thrombisis
GCP: good clinical practice
GCS: graduated compression stockings
LMWH: low-molecular-weight heparin
VTE: venous thromboembolism.

## GENERAL INFORMATION

Protocol Title: Intraoperative intermittent pneumatic compression (IPC) reduces incidence of venous thromboembolism (VTE) in patients undergoing craniotomy: Study protocol of a randomized multicenter, single-blind trial.

Registry: German Clinical Trials Register (Deutsches Register Klinischer Studien): DRKS00034190.

Study Dates: June 2024 to June 2027.

Sponsor/Funding Agency: German Research Foundation (Deutsche Forschungsgemeinschaft); Project number 491399287.

Institutional Approvals: Institutional Review Board (Ethics Committee, Faculty of Medicine of the Martin Luther University Halle-Wittenberg, Processing number: 2023-243).

### Roles and Responsibilities

Principal investigator: apl. Prof. Dr med. Julian Prell.

Statistician: apl. Prof. Dr Andreas Wienke.

Co-investigators: PD Dr med. Stefan Rampp, Dr med. Maximilian Scheer.

## RATIONALE AND BACKGROUND INFORMATION

Venous thromboembolism (VTE) subsumes deep vein thrombosis (DVT) and pulmonary embolism (PE). Compared with other patient groups, neurosurgical patients are considered to be at an increased risk for these conditions. This is due to presence of malignancy, long duration of procedures, reduced mobility, and direct release of procoagulants such as tissue factors from brain tissue.^1-3^ Up to 50% of patients develop VTE after craniotomy.^4,5^ In the majority of cases, this condition is asymptomatic. However, symptomatic VTE occurs in 7.5% of patients undergoing craniotomy,^6-8^ and even asymptomatic VTE localized to distal muscle veins can progress to symptomatic deep vein thrombosis.^9-13^ This can lead directly and without clinical warning to PE in 7% of affected patients.^14^ Such a PE is caused by DVT in >90% of cases,^15^ occurs in 3.7% of all patients undergoing craniotomy, and is fatal in up to 50% of all affected neurosurgical patients.^5,13,16^ Thus, sufficient prophylaxis of VTE would considerably reduce unnecessary additional risks associated with neurosurgical procedures. Two different approaches have been described to achieve this: pharmacological prophylaxis, eg, by low-molecular-weight heparin (LMWH) or unfractionated heparin on the one hand, and mechanical prophylaxis by graduated compression stockings (GCS) or IPC on the other hand. With IPC, multichambered sleeves are positioned around the lower extremity and sequentially inflated to “milk” the blood from the veins, mimicking the physiological muscle pump. Most trials studying the effect of IPC have implemented this method for prolonged time intervals of several days after surgery; as a consequence of these long time intervals, poor compliance in using the devices has been reported as an issue in several publications.^17,18^

## STUDY GOALS AND OBJECTIVES

The main objective is:

Evaluation of the intraoperative use of IPC to reduce the incidence of VTE in patients undergoing craniotomy.

## STUDY DESIGN

Prospective, randomized, single-blinded multicenter trial.

### Inclusion Criterion

- Elective surgery for intracranial pathology via craniotomy.

### Exclusion Criteria

- Gravidity
- Age <18 years
- Perioperative administration of blood products
- Abnormal blood coagulation (thrombocytes and/or plasmatic coagulation, liver disease, antiplatelet agents)
- Local leg condition with which the sleeves may interfere, such as dermatitis, vein ligation gangrene, recent skin graft, severe arteriosclerosis, or other ischemic vascular disease
- Massive edema of the legs or pulmonary edema from congestive heart failure
- Extreme deformity of the leg
- Preexisting thromboembolism
- Clinical signs of deep vein thrombosis

### Sample Size

**FIGURE 1.**
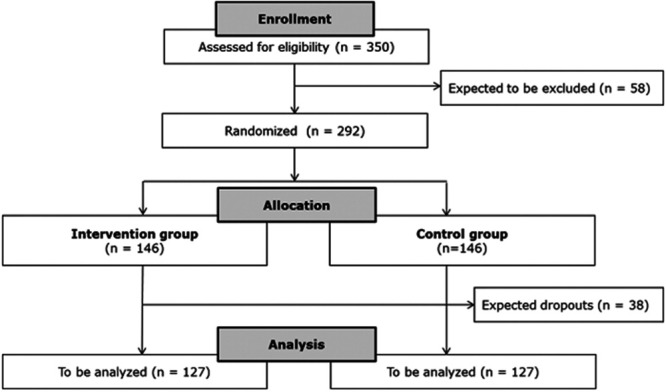
Flowchart of the study.

Sample size calculation for the comparison of the VTE rates in the treatment and control groups is based on a two-sided chi-square test with significance level alpha of 5% and a power of 90%. The pilot study^13^ showed a VTE incidence of 26% in the control group vs 7% in the treatment group. To avoid overoptimistic treatment effect assumptions, we assume VTE rates of 9% and 24% in the treatment and control groups, respectively. This results in 127 patients per treatment group. Assuming a similar dropout rate as in the previous study (approx. 13%), 146 patients per group (292 in total) will have to be recruited (Figure 1).

The analysis of the primary end point will be performed by logistic regression using treatment group and treatment center as covariates because of the stratified randomization with respect to treatment center. The logistic regression analysis provides more power compared with the chi-square test mentioned above. Therefore, the planned sample size calculation is conservative.

An interim analysis of the 2 VTE rates will be performed after recruitment of 50% of patients (n = 146) to adapt sample size if necessary. With respect to the specific history of this scientific project, we assign a high priority to the exclusion of underpowering. The intention of the interim analysis is not to stop the trial early because of futility or efficacy.

- To be assessed for eligibility (n = 350).
- To be assigned to the trial, ie, recruited (n = 292).
- To be analyzed (n = 254).

## METHODOLOGY

### Control(s)/Comparator(s)

The control group is treated according to standards of clinical routine. Thus, the incidence of VTE in this group should reflect the status quo in surgical therapy of patients harboring intracranial pathology. Patients in the treatment group are treated exactly the same way with the sole exception of intraoperative administration of IPC. Consequently, any difference in incidence of VTE between both groups should be attributable to IPC.

### Study Population

Patients undergoing craniotomy are at significant risk for VTE, which can hypothetically be lowered by IPC. Thus, undergoing elective surgery for intracranial pathology was chosen as the inclusion criterion. On the one hand, exclusion criteria were chosen to exclude patients in whom VTE incidence might be influenced by additional pathology (eg, spontaneous or iatrogenic disturbances in blood coagulation); on the other hand, patients with conditions that represent contraindications for IPC (according the manufacturer of the device and current guidelines) are to be excluded.

### Treatments/Procedures

All patients will receive GCS perioperatively and LMWH starting on the first postoperative day. Both groups will receive IPC sleeves for the complete duration of the surgical procedure. The sleeves will be put on by trained personnel during positioning of the patient; they are standard, CE-certified equipment, which is specifically approved for use in the prevention of VTE. The device Flowtron ACS900 (Arjo) will be used in all patients. In the *control group*, it will not be activated; otherwise, they are treated exactly the same way.

### Additional Treatments

In case of evidence of thrombosis or PE, drug therapy is initiated according to general practice guidelines as established by the respective center. If thrombosis is detected, compression therapy is also started accordingly.

### Outcome Measures

- Primary end point:
  Incidence of VTE
- Secondary end points:
  Proximal and distal deep vein thrombosis, symptomatic vs asymptomatic VTE, muscle vein thrombosis, central venous catheter–related thrombosis, PE, death, influence of lesion type, and duration of surgery.

### Data Collection

The Coordination Center for Clinical Trials will be responsible for data management and data archiving. The data and essential trial sponsor documents (Master File) will be managed and archived according to existing standards and regulations, in a data format allowing long-term preservation and future reuse. The sites will also be instructed to archive respective essential documents (investigator site file, CRFs, and source data) according to existing standards and regulations. The trial is registered with full description at the German Clinical Trials Register (Deutsches Register Klinischer Studien, DRKS).

## DISCUSSION

IPC exclusively during the surgical procedure and in addition to standard prophylaxis consisting of GCS and LMWH would avoid such problems. In a pilot study,^13^ we evaluated such a novel shortened IPC regimen. The results showed a statistically significant risk difference of 19.1% [95% CI 5.9%-62.1%] and number needed to treat of 5.24 between patients with additional intraoperative IPC and patients only receiving standard prophylaxis. Despite randomization, a significant difference between duration of surgery in the groups limited the validity of this result. The study was underpowered to compensate for this issue.

As the larger number of patients needed for the new trial exceeds the capabilities of a single center, we designed a multicentric trial with financial support by German Research Foundation (Deutsche Forschungsgemeinschaft). If by this trial intraoperative IPC is confirmed to reduce the risk for VTE on the scale observed in our pilot study, the potential benefit for neurosurgical patients would be considerable. The results would potentially influence treatment guidelines in providing the needed high-quality evidence for robust recommendations.

## TRIAL STATUS

The current status of the trial is in the preimplementation stage.

## SAFETY CONSIDERATIONS

### Assessment of Safety

Adverse events associated with IPC will be monitored and reported according to good clinical practice (GCP) principles and regulatory requirements. Assessment of safety does not constitute an end point of this study.

Our pilot study indicated that application of IPC during the surgical procedure is safe, and that it reduces VTE risk considerably.^18^ Previous studies have not reported any severe complications by use of IPC. For the proposed additional, exclusively intraoperative use, there is currently no evidence. The procedure is therefore not considered standard practice. Thus, it is not unethical to withdraw IPC from patients of the control group, which will receive medical care according to local standards. In addition, even patients of the control group will benefit from improved detection rates of VTE by the Doppler sonography each patient will receive (not part of standard clinical practice). Evidence on the efficacy of the easy-to-implement regimen would, however, significantly affect clinical care and ultimately benefit patients. An independent data safety monitoring board will closely review safety data on a regular basis and will make recommendations regarding further conduct of the trial, eg, to continue or hold enrollment, to amend protocol, or to stop the study early.

## FOLLOW-UP

### Follow-up per Patient

Doppler sonography will be performed within the first 7 postoperative days.

### Duration of Intervention per Patient

All patients (both treatment and control groups) are treated with GCS from patient positioning until the fifth postoperative day, combined with subcutaneous administration of low-molecular-weight heparin, which is started with the first postoperative day and continued until discharge.

All trial-related investigations (intervention and follow-up) are limited to the single stay of each patient for their surgery. During the surgical procedure, IPC is applied. The primary end point (incidence of VTE) is then evaluated by Doppler sonography within 7 days after surgery (Table). No further follow-up is planned.

**TABLE.** Trial-Related Investigations

Time point	Trial measure
Perioperatively	Graduated compression stockings
Surgical procedure	IPC (activated/not activated)
Starting on first postsurgical day	LMWH according to the specific standards of the respective center
Surgery to 7th postoperative day	Doppler-sonography: primary (incidence of VTE) and secondary end points
Surgery to 7th postoperative day	Assessment of safety

IPC, intermittent pneumatic compression; LMWH, low-molecular-weight heparin; VTE, venous thromboembolism.

## DATA MANAGEMENT AND STATISTICAL ANALYSIS

### Methods Against Bias

Bias in assessment is addressed by a randomized single-blinded study design. Randomization will be stratified with respect to treatment center and will be performed centrally via an internet-based automatic system at the local Coordination Centre for Clinical Trials. The personnel performing Doppler sonography and diagnosis of PE will be blinded to group status of all patients; in addition, sonography will be performed according to Deutsche Gesellschaft für Ultraschall in der Medizin guidelines (Untersuchung der tiefen Beinvenen und Beckenvenen, Grundlagen und Technik).^19^ All ultrasound examiners involved in the study will meet and establish consensus on the definitions of DVT and its subtypes before the study starts. Patients are blinded to the randomization. To ensure this and to enable identical treatment in all patients of the treatment group, the same type of IPC device (Flowtron ACS900 (Arjo)) will be used in all centers taking part in the study. To avoid a center bias, all centers participating in the study will be limited to a maximum number of 100 patients over the duration of the study. However, the centers will be instructed to recruit their patients from a consecutive series to not introduce selection bias. As a consequence of this concept, there is a limit of 100 patients over the duration of the study per center despite the obvious ability of certain centers to contribute a higher number of patients.

The study will be analyzed according to the intention-to-treat principle to avoid attrition bias. It is registered prospectively in the DRKS (German Register of Clinical Studies; DRKS00034190) and will be reported according to CONSORT (Consolidated Standards of Reporting Trials).

### Statistical Analysis

The analysis of the primary end point will be performed by logistic regression using treatment group and treatment center as covariates because of the stratified randomization with respect to treatment center based on the intention-to-treat population. A per-protocol analysis will serve as sensitivity analysis. In addition, according to the very important covariate “duration of surgery” in the previous study^18^ (which could not be used as a stratification variable), an additional logistic regression using treatment group, treatment center, and duration of surgery as covariates will be performed as sensitivity analysis as well.

Analysis of binary secondary end points will be performed in the same way. All secondary variables will be analyzed in an exploratory manner. An interim analysis of the 2 VTE rates will be performed after recruitment of 50% of patients to adapt sample size if necessary (without analyzing futility or efficacy).

### Access to Data

Access to data set will be made available on request.

### Dissemination Policy

The results of the trial will be published according to CONSORT guidelines in peer-reviewed national and international journals, with special emphasis on media relevant to neurosurgeons and angiologists, regardless of the size or direction of the effects. In addition to regular publication in peer-reviewed journals, results will be presented and discussed at national and international neurosurgical and vascular congresses. Data will also be published in the relevant publications of patient support organizations. To increase patient interest and support, we will provide information in lay language through specific publications on our websites, printed flyers, and regular oral presentations at local and national meetings of patient organizations in Germany.^20^

## QUALITY ASSURANCE

The coordinating investigator is responsible for implementing and maintaining quality assurance and quality control systems with written standard operating procedures (SOPs) to ensure that the trial is conducted and the data generated, documented, and reported in accordance with the protocol, GCP, and applicable regulatory requirements.^21^ Monitoring and data reviewing will be done according to a risk-adapted approach by the local Coordination Center for Clinical Trials, which has comprehensive experience managing clinical trials. Tasks assigned to this center will be done according to the written SOPs and German-wide Koordinierungszentrum für klinische Studien Network. Monitoring will be performed by independent CRAs (Coordination Center for Clinical Trials) according to International Council for Harmonisation of Technical Requirements for Registration of Pharmaceuticals for Human Use-GCP E6 and SOP to ensure patient safety, adherence to protocol, and consistency of the data. The monitoring will be done centrally (check of the data entered into the eCRFs) and by means of on-site visits at the respective study center.

Pretrial visits will be carried out by CRAs to assess the suitability of the trial site in terms of facilities and the qualifications and availability of the trial personnel. Based on the prestudy visits, the coordinating investigator will decide whether the site will participate in the study. Teleconferences (each site separately) will be held to coordinate the site prestudy visits for site initiation. The initiation will be supported by Power Point presentations and study materials (investigator's site file, protocol, informed consent, CRFs, etc.) that will be provided to the sites in advance. On-site monitoring at each site is planned by means of regular visits from the beginning to the end of the trial to check the completeness of the patient records, the consistency of the entries in the CRFs, and the compliance with the protocol and GCP. The specific scope of monitoring and source data verification will be specified in the monitoring manual. Each trial site will be monitored shortly after the first subject is enrolled and treated.^22^ Early monitoring will ensure prompt intervention by the clinical research associate or prinicipal investigator in case of problems at the site, such as major inconsistencies in study conduct and CRF completion. A further 7 (average, individual risk-based number depending on recruitment rate and quality of CRF completion) monitoring visits will be made to each site during the enrollment period.^23^ In addition, regular central data monitoring via electronic CRFs will be performed throughout the study to ensure ongoing data quality. Each study site will undergo a close-out visit by the clinical research associate after the last participant at that site has completed follow-up. To ensure data quality and study conduct, the sponsor may conduct site visits by an independent auditor.^24^

## EXPECTED OUTCOMES OF STUDY

Reduction of incidence of VTE.

## DURATION OF THE PROJECT

First patient in to last patient out (months): 24.

Duration of the entire trial (months): 36.

Recruitment period (months): 27.

**FIGURE 2.**
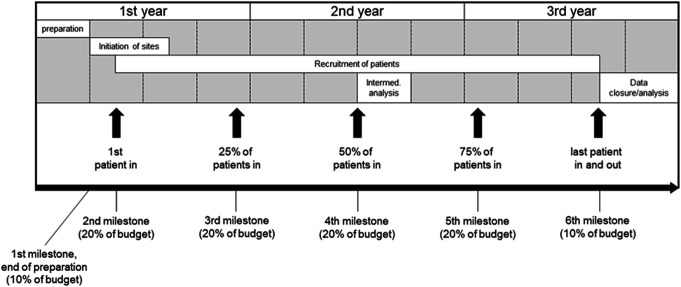
Timeline of the study.

A timeline of the study is shown in Figure 2.

## ETHICS

This study will be performed in accordance with the Declaration of Helsinki. A positive ethics vote in favor of conducting the study has been received (Bearbeitungsnummer: 2023-243). All patients are informed in detail beforehand and must provide written consent before participating.

### Data Availability

All data will be made available upon request.
